# The Basic Leucine Zipper Transcription Factor PlBZP32 Associated with the Oxidative Stress Response Is Critical for Pathogenicity of the Lychee Downy Blight Oomycete Peronophythora litchii

**DOI:** 10.1128/mSphere.00261-20

**Published:** 2020-06-03

**Authors:** Guanghui Kong, Yubin Chen, Yizhen Deng, Dinan Feng, Liqun Jiang, Lang Wan, Minhui Li, Zide Jiang, Pinggen Xi

**Affiliations:** a Department of Plant Pathology, Guangdong Province Key Laboratory of Microbial Signals and Disease Control, South China Agricultural University, Guangzhou, China; b Integrative Microbiology Research Centre, South China Agricultural University, Guangzhou, China; c Guangdong Province Key Laboratory of New Technology in Rice Breeding, Rice Research Institute, Guangdong Academy of Agricultural Sciences, Guangzhou, China; University of Georgia

**Keywords:** *Peronophythora litchii*, bZIP transcription factor, pathogenicity, peroxidase, laccase

## Abstract

Basic leucine zipper (bZIP) transcription factors are widespread in eukaryotes, including plants, animals, fungi, and oomycetes. However, the functions of bZIPs in oomycetes are rarely known. In this study, we identified a bZIP protein possessing a special bZIP-PAS structure in Peronophythora litchii, named *PlBZP32*. We found that *PlBZP32* is upregulated in zoospores, in cysts, and during invasive hyphal growth. We studied the functions of *PlBZP32* using the RNAi technique to suppress the expression of this gene. *PlBZP32*-silenced mutants were more sensitive to oxidative stress, showed a lower cyst germination rate, and produced more sporangia than the wild-type strain SHS3. The *PlBZP32*-silenced mutants were also less invasive on the host plant. Furthermore, we analyzed the activities of extracellular peroxidases and laccases and found that silencing *PlBZP32* decreased the activities of *P. litchii* peroxidase and laccase. To our knowledge, this is the first report that the functions of a bZIP-PAS protein are associated with oxidative stress, asexual development, and pathogenicity in oomycetes.

**IMPORTANCE** In this study, we utilized the RNAi technique to investigate the functions of PlBZP32, which possesses a basic leucine zipper (bZIP)-PAS structure, and provided insights into the contributions of bZIP transcription factors to oxidative stress, the production of sporangia, the germination of cysts, and the pathogenicity of Peronophythora litchii. This study also revealed the role of PlBZP32 in regulating the enzymatic activities of extracellular peroxidases and laccases in the plant-pathogenic oomycete.

## Abstract (precis)

In this study, we utilized the RNAi technique to investigate the functions of PlBZP32, which possesses a basic leucine zipper (bZIP)-PAS structure, and provided insights into the contributions of bZIP transcription factors to oxidative stress, the production of sporangia, the germination of cysts, and the pathogenicity of Peronophythora litchii. This study also revealed the role of PlBZP32 in regulating the enzymatic activities of extracellular peroxidases and laccases in the plant-pathogenic oomycete.

## INTRODUCTION

Lychee (or litchi; Litchi chinensis Sonn.) is a famous tropical and subtropical fruit with high economic value due to its appearance, taste, and nutrition, and its production is approximately 3.5 million tons worldwide each year (1, 2). Downy blight caused by Peronophythora litchii is one of the most destructive diseases of lychee during production and postharvest (3, 4).

In plant-microbe interactions, one of the most rapid plant defense reactions is the oxidative burst, which constitutes the production of reactive oxygen species (ROS), including superoxide and H_2_O_2_ generated by NADPH oxidases (5, 6). The accumulation of ROS at the site of pathogen invasion can either directly kill the pathogen (7, 8) or function as a second messenger to induce the expression of various plant defense-related genes and to trigger pathogen-associated molecular pattern (PAMP)-triggered immunity (PTI) in plants (9–12).

The transcription factors (TFs) of the basic leucine zipper (bZIP) family are multifunctional across pathogenic fungi. For example, MoAtf1 regulates the transcription of genes encoding laccases and peroxidases, the oxygen scavengers, and thus is required for full virulence in the rice blast fungus Magnaporthe oryzae (13). MoAP1 also mediates the oxidative stress response and is critical for growth, conidium formation, and pathogenicity in *M. oryzae* (14). In another plant-pathogenic fungus, Fusarium oxysporum, HapX mediates iron homeostasis; therefore, it is essential for rhizosphere competence and virulence of this pathogen (15). In the human pathogen Cryptococcus neoformans, Gsb1 is required for oxidative stress response, mating, and virulence (16). The Yap1-involved H_2_O_2_ detoxification is also associated with virulence in Ustilago maydis (17), Candida albicans (18), Alternaria alternata (19), Verticillium dahliae (20), Aspergillus parasiticus (21), and Colletotrichum gloeosporioides (22). However, in Cochliobolus heterostrophus and Aspergillus fumigatus, CHAP1 and AfYap1 are associated with the sensitivity against H_2_O_2_ and menadione but are not essential for virulence (23, 24).

Oomycetes, which include many notorious plant pathogens, are fungus-like organisms; however, they are evolutionarily related to brown algae and belong to the kingdom Stramenopila (25). Systematic analysis identified conventional bZIPs and novel bZIP transcription factors in Phytophthora infestans (26), Phytophthora ramorum, and Phytophthora sojae (27, 28). Several bZIPs from *P*. *sojae* are upregulated by H_2_O_2_ treatment (28). Using stable gene silencing analyses, several *P*. *infestans* bZIPs were found to play roles in protecting the pathogen from hydrogen peroxide-induced injury (26). The novel *P. infestans* bZIP PITG_11668 was nucleus localized, suggesting that it is an authentic transcription factor (26). Pibzp1 from *P. infestans* was functionally characterized, which interacts with a protein kinase and is required for zoospore motility and plant infection (29).

A Per-ARNT-Sim (PAS)-containing bZIP transcription factor was annotated in *P. sojae*, named PsBZPc32 (28), but without functional characterization. In this study, we identified the PsBZPc32 ortholog in *P*. *litchii* named PlbZIP32. We analyzed the sequences of BZP32 orthologs, as well as the transcriptional profile of *PlBZP32*. We further characterized the function of the *PlbZIP32* gene in oxidative stress response, asexual sporulation, and pathogenicity. Our results showed that *PlBZP32* is required for the asexual development, oxidative stress response, and pathogenicity of *P*. *litchii*.

## RESULTS

### *PlBZP32* gene belongs to a bZIP transcription factor family and is upregulated in zoospores, cysts, and late stages of infection.

**FIG 1:**
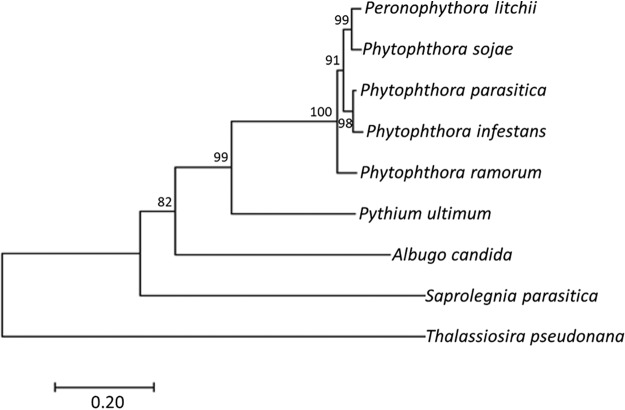
Phylogenetic analysis of PlBZP32 protein and its orthologs. The evolutionary history was inferred using the neighbor-joining method (47). Evolutionary analyses were conducted in MEGA7 (48).

*P. litchii BZP32* (*PlBZP32*) is the ortholog of *P. sojae BZPc32*, which encodes a bZIP transcription factor and possess a unique bZIP-PAS structure (30). There are 50 bZIP transcription factors in *P*. *litchii* based on a search with the Batch CD-search tool (CDD; in NCBI) (31), while PlBZP32 is the only one containing a bZIP-PAS structure (see Table S1 in the supplemental material). Here, we also searched the orthologous proteins from *P. infestans*, P. parasitica, *P. ramorum*, Pythium ultimum, Albugo candida, Saprolegnia parasitica, and Thalassiosira pseudonana for sequence alignment and phylogenetic analysis. PlBZP32 is a 392-amino acid (aa) protein with a bZIP domain located in aa positions 246 to 294 and a PAS domain located in aa positions 304 to 376 (see Fig. S1 in the supplemental material). We found that the bZIP domain-PAS structure is conserved in all the orthologs examined here (Fig. S1). Phylogenetic analysis showed that these kinds of bZIP transcription factors are widespread and conserved in oomycetes and algae, while Thalassiosira pseudonana is outside the oomycete group (Fig. 1).

10.1128/mSphere.00261-20.1 TABLE S1 Sequences of *P. litchii* bZIP transcription factors. Download Table S1, XLSX file, 0.05 MB. Copyright © 2020 Kong et al. 2020 Kong et al. This content is distributed under the terms of the Creative Commons Attribution 4.0 International license.

10.1128/mSphere.00261-20.3 FIG S1 Amino acid sequence alignment of PlBZP32 and its orthologs from *P*. *sojae*, *P*. *parasitica*, *P*. *ramorum*, *P*. *infestans*, *Pythium ultimum*, *Albugo candida*, *Saprolegnia parasitica*, and *Thalassiosira pseudonana*. The dark red and blue boxes represent bZIP domains and PAS domains, respectively. Download FIG S1, JPG file, 1.0 MB. Copyright © 2020 Kong et al. 2020 Kong et al. This content is distributed under the terms of the Creative Commons Attribution 4.0 International license.

**FIG 2:**
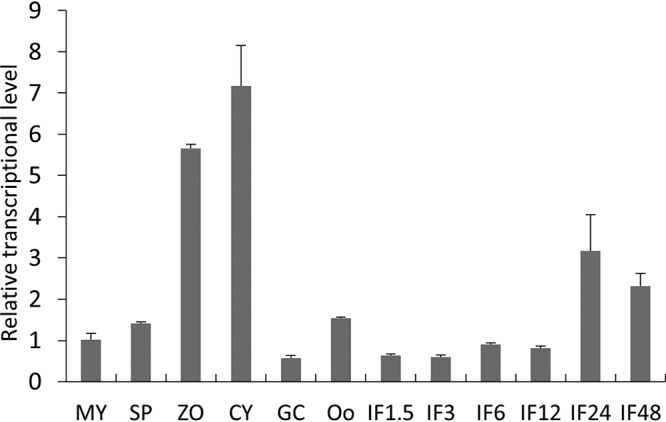
Transcriptional proﬁle of the *PlBZP32* gene. Relative transcriptional levels of *PlBZP32* were determined by qRT-PCR with total RNA extracted from speciﬁc stages of the life cycle. MY, mycelia; SP, sporangia; ZO, zoospores; CY, cysts; GC, germinating cysts; Oo, oospores; IF1.5 to IF48, infection stages of 1.5, 3, 6, 12, 24, and 48 h postinoculation. Transcriptional levels were normalized using the MY values as “1,” and the *P*. *litchii ACTIN* gene served as an internal control. The experiment was repeated three times with independent samples.

To investigate the biological function of *PlBZP32*, we first examined the transcriptional profile of the *PlBZP32* gene in various growth stages, including mycelia, sporangia, zoospores, cysts, germinating cysts, and oospores, and in infection stages, including 1.5, 3, 6, 12, 24, and 48 h postinoculation. Our results showed that *PlBZP32* was upregulated in zoospores, cysts, and late stages of infection compared with that of the vegetative mycelial growth stage (Fig. 2), suggesting that PlBZP32 might function in these specific stages.

### Generation of *PlBZP32*-silenced transformants.

**FIG 3:**
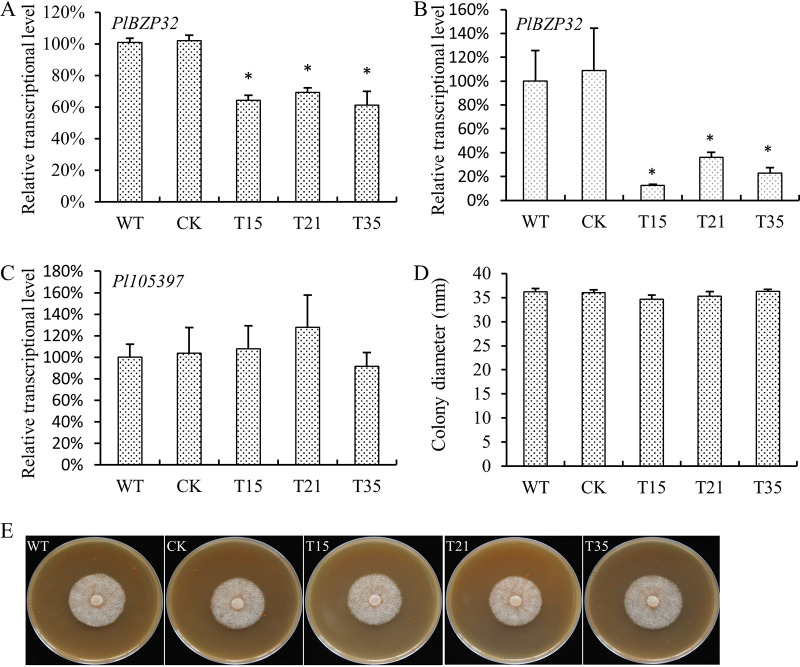
*PlBZP32* was not required for the mycelial growth of *P*. *litchii*. (A, B) Relative transcriptional levels of *PlBZP32* in wild type (WT), CK (transformed with empty vector), and transformants. *PlBZP32* transcription was normalized to that of the WT value (set as “1.0”). The *P*. *litchii ACTIN* gene served as an internal control. RNA used in qRT-PCR was extracted from mycelium (A) and zoospore (B). (C) Transcriptional level of *Pl105397* in WT, CK, and *PlBZP32*-silenced transformants. (D) Colony diameter of WT, CK, and the three *PlBZP32*-silenced transformants. Bar charts A to D depict means ± SD derived from 3 independent repeats, each of which contained 3 replicates. Asterisks on the bars denote significant difference (*P < *0.05) based on Duncan’s multiple range test method. (E) WT, CK, and the *PlBZP32*-silenced transformants were inoculated on CJA medium for 3 days at 28°C in darkness and then photographed. These experiments were repeated three times.

To generate the *PlBZP32*-silenced mutants, we used a polyethylene glycol (PEG)-mediated protoplast transformation with a pTORmRFP4 vector carrying an antisense full-length copy of *PlBZP32*. We obtained 182 transformants by antibiotic resistance screening. These transformants were confirmed by genomic PCR and subsequent quantitative reverse transcription PCR (qRT-PCR). Among them, three transformants (T15, T21, and T35) showed significant reduction in *PlBZP32* transcriptional levels in mycelium and zoospore, compared with the wild-type strain SHS3 and the CK strain in which the backbone vector pTORmRFP4 was transformed (Fig. 3A and B). Considering that homology-based transcriptional silencing might cause off-target effects, especially those affecting the expression of neighboring gene(s) (32, 33), we searched the neighboring locus of *PlBZP32* and found one gene (*Pl105397*; GenBank accession number MT396990) 875 nucleotides (nt) away from *PlBZP32*. *Pl105397* encodes a structural maintenance of chromosomes (SMC) protein containing a Discs-large homologous regions (DHR) domain. The transcriptional level of *Pl105397* in *PlBZP32*-silenced transformants was also examined, and no significant difference was achieved compared with wild-type and CK strains (Fig. 3C).

We checked the growth of these *PlBZP32*-silenced transformants in comparison with wild-type and CK strains. We measured the colony diameter of these strains at 3 days after inoculation and found that the silencing of the *PlBZP32* gene did not affect the growth of *P*. *litchii* when cultured on carrot juice agar (CJA) medium (Fig. 3D and E).

### *PlBZP32*-silenced mutants were more sensitive to oxidative stress.

**FIG 4:**
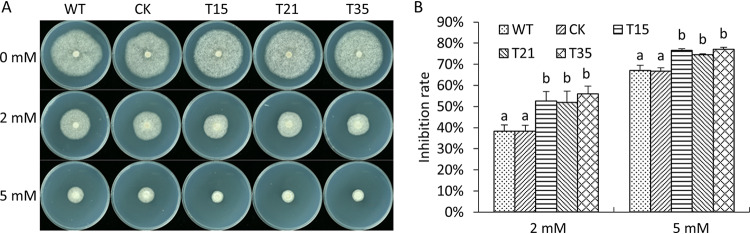
The *PlBZP32*-silenced mutants were hypersensitive to H_2_O_2_. (A) The wild type (WT), CK, and *PlBZP32*-silenced mutants were inoculated on Plich medium with or without 2 or 5 mM H_2_O_2_ and cultured at 25°C for 7 days. (B) The colony diameters of the tested strains were measured. Wild-type and CK strains were used as controls. The growth inhibition rate was calculated using the following formula: (the diameter of control − the diameter of treated strain)/(the diameter of control) ×100%. The bar chart depicts means ± SD derived from 3 independent repeats, and different letters represent a significant difference (*P < *0.05) based on Duncan’s multiple range test method.

To investigate whether PlBZP32 is involved in the response to oxidative stress, the *PlBZP32*-silenced mutants and wild-type strain were exposed to H_2_O_2_ concentrations of 2 and 5 mM, respectively. The *PlBZP32*-silenced mutants displayed a greater growth inhibition rate than the wild-type and CK strains (Fig. 4). These results suggested that *PlBZP32* is required for resistance to oxidative stress.

**FIG 5:**
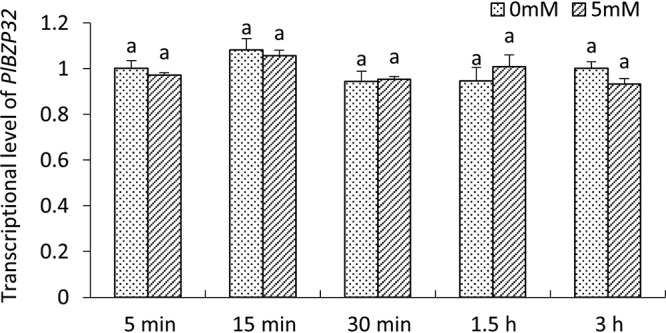
Transcription of *PlBZP32* during H_2_O_2_ treatment. *P*. *litchii* mycelia were treated with 5 mM H_2_O_2_ for 5 min, 15 min, 30 min, 1.5 h, and 3 h, before collection for total RNA extraction and qRT-PCR analysis. Expression of the *PlBZP32* gene in the untreated mycelia collected at 5 min was used as a reference and normalized to 1.0. The bar chart depicts means ± SD derived from 3 independent repeats. The same letter on the top of bars represents no signiﬁcant difference (*P > *0.05), based on Duncan’s multiple range test method.

We also analyzed the transcription levels of *PlBZP32* after treatment with 5 mM H_2_O_2_. We found no significant changes in the transcription level of *PlBZP32* from 5 minutes to 3 h posttreatment, compared with the untreated control (Fig. 5). These results suggests that the involvement of PlBZP32 in oxidative stress management might be not associated with a transcription-level regulation of this gene.

### Silencing of *PlBZP32* impaired the pathogenicity of *P*. *litchii*.

**FIG 6:**
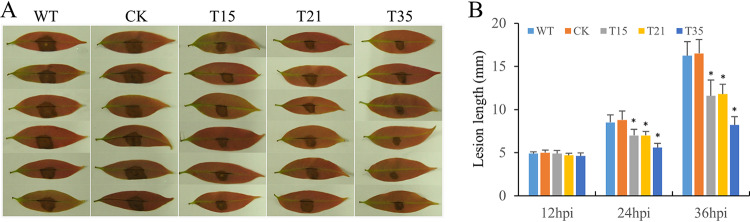
*PlBZP32* was required for full virulence of *P*. *litchii*. (A) Wild type (WT), CK, and three *PlBZP32*-silenced mutants (T15, T21, and T35) were inoculated on lychee leaves and kept at 25°C in the dark. Photographs were taken 36 h postinoculation (hpi). Representative images for each instance were displayed. (B) Lesion length was measured at 12, 24, and 36 hpi. The values are means ± SD derived from three independent biological repeats (*n* = 12 leaves for each strain). Asterisks denote a significant difference (*P < *0.05) based on Duncan’s multiple range test method.

The production and accumulation of reactive oxygen species, the oxidative burst, has been shown to occur in plant-pathogen interactions, such as H_2_O_2_ and O^2−^, directly acting as antimicrobial agents (34, 35). Given that silencing of *PlBZP32* decreased the resistance of *P*. *litchii* to H_2_O_2_, we next analyzed whether *PlBZP32* is involved in the pathogenicity of this pathogen. *PlBZP32*-silenced mutants and the wild-type strain were inoculated on lychee leaves. At 12, 24, and 36 h after inoculation, we measured the diameter of lesions. The results showed that the lesions caused by *PlBZP32*-silenced mutants were significantly smaller than that caused by wild-type and CK strains (Fig. 6), indicating that *PlBZP32* is required for the full pathogenicity of *P*. *litchii*.

### Silencing of PlBZ*P32* affected the production of sporangia and germination of cysts.

**FIG 7:**
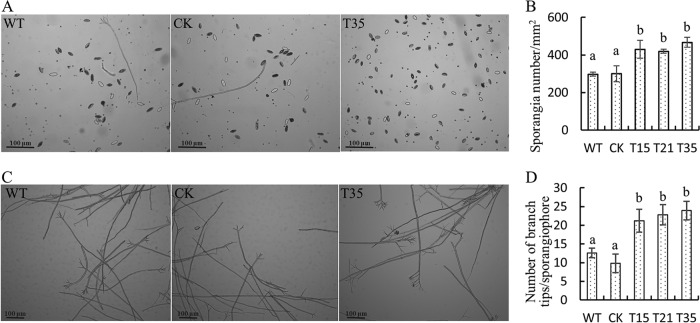
*PlBZP32*-silenced mutants produced more sporangia. (A, B) Sporangia were quantified at 5 days after inoculation. Bar chart represents mean ± SD (C, D). Branch tips of each sporangiophore were counted. These results are derived from three independent biological repeats, each of which contained 5 technical replicates. Different letters represent a significant difference (*P < *0.05) based on Duncan’s multiple range test method.

**FIG 8:**
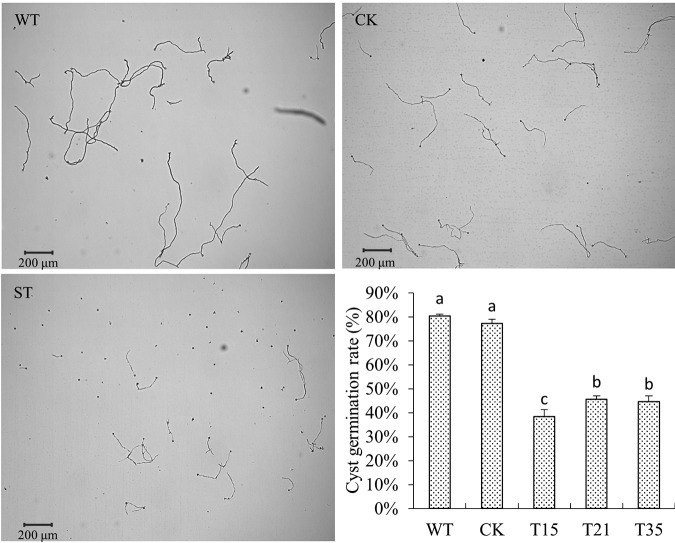
Silencing of *PlBZP32* impaired cyst germination of *P*. *litchii*. Cyst germination was quantified at 2 h after cyst formation. In the bar graph, mean values present the cyst germination rates of *PlBZP32*-silenced mutants or control strains. Different letters represent a significant difference (*P < *0.05) based on Duncan’s multiple range test method. This experiment was repeated three times. Scale bars, 200 μm.

Transcriptional profiling analyses showed that the *PlBZP32* gene was upregulated in asexual stages (zoospore and cyst) of *P*. *litchii*, so we also analyzed the production of sporangia and the germination rate of cysts. Our results showed that *PlBZP32*-silenced mutants produced approximately 40% to 50% more sporangia than wild-type and CK strains (Fig. 7A and B) and each sporangiophore of *PlBZP32*-silenced mutants produced more branch tips and sporangia (Fig. 7C and D). We tested the germination rate of cysts and found it was significantly decreased in the *PlBZP32*-silenced mutants. The germination rate of cysts is less than 50% in *PlBZP32*-silenced mutants, whereas it was approximately 80% in wild-type or nonsilenced strains (Fig. 8). However, the silencing of *PlBZP32* did not significantly affect the length and germination of sporangia (see Fig. S2 and S3 in the supplemental material). Thus, *PlBZP32* may negatively regulate the sporangium production and positively regulate the cyst germination.

10.1128/mSphere.00261-20.4 FIG S2 Silencing of PlBZP32 did not affect the sporangia. The length of sporangia was measured; there is no statistically significant difference (*P* > 0.05) using the Student’s *t* test. The bar chart represents mean ± SD derived from three independent biological repeats. Download FIG S2, TIF file, 0.1 MB. Copyright © 2020 Kong et al. 2020 Kong et al. This content is distributed under the terms of the Creative Commons Attribution 4.0 International license.

10.1128/mSphere.00261-20.5 FIG S3 PlBZP32 is not required for sporangium germination of *P. litchii*. The sporangium germination was calculated 2 hours after sporangia were collected. Bar chart represents mean ± SD derived from three independent biological repeats. Download FIG S3, TIF file, 0.2 MB. Copyright © 2020 Kong et al. 2020 Kong et al. This content is distributed under the terms of the Creative Commons Attribution 4.0 International license.

### *PlBZP32* disruption attenuates the activities of extracellular peroxidases and laccases.

Peroxidase and laccase have been reported to be involved in the resistance of ROS and, thus, critical for fungal pathogenicity (14, 36). Given that the *PlBZP32*-silenced mutants displayed reduced pathogenicity compared with the wild-type (WT) and nonsilenced strains, we further analyzed the peroxidase and laccase activities in these strains.

**FIG 9:**
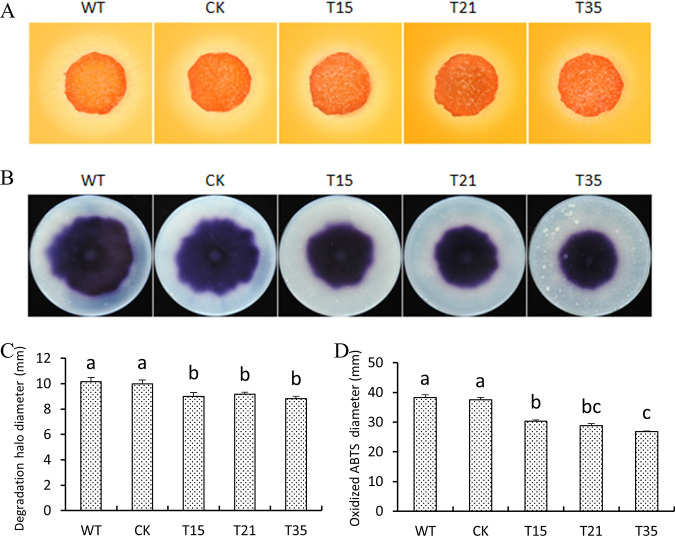
*PlBZP32* regulated activities of extracellular peroxidases and laccases. (A) Peroxidase activity assay. Mycelial mats of WT, CK, and three *PlBZP32*-silenced transformants were inoculated onto solid Plich medium containing Congo red at a ﬁnal concentration of 500 μg/ml, as an indicator. The discoloration of Congo red was observed after incubation for 1 day. (B) Laccase activity assay. Mycelial mats of the aforementioned strains were inoculated on lima bean agar (LBA) media containing 0.2 mM ABTS for 10 days. (C) The discoloration halo diameters were measured at 1 day postinoculation. (D) The diameters of oxidized ABTS (dark purple) were measured at 10 days postinoculation. Different letters in (C) and (D) bar charts represent a significant difference (*P < *0.05) based on Duncan’s multiple range test method. These two experiments contains three independent biological repeats, each of which contained three replicas.

Extracellular peroxidase activity was assessed based on Congo red (CR) degradation (37). As shown in Fig. 9A and C, diameters of the degradation halos caused by the three *PlBZP32*-silenced mutants were reduced compared with the controls. On the other hand, the activities of the extracellular laccase were measured by an oxidation assay of ABTS [2,2′-azinobis (3-ethylbenzothiazolinesulfonic acid)] (37). Three *PlBZP32*-silenced mutants showed a significantly decreased accumulation of ABTS, as visualized by dark purple staining around the mycelial mat, compared with the nonsilenced and wild-type strains (Fig. 9B and D).

## DISCUSSION

In oomycetes, bZIP transcription factors are a large protein family. Fifty bZIP transcription factors were identified in *P*. *litchii*; 71 bZIP transcription factors were predicted from *P*. *sojae*, of which 45 were confirmed by CDD (NCBI conserved domain database) or the SMART database (28); 22 bZIPs were identified in *P*. *ramorum* (27); and 38 bZIPs were identified in *P*. *infestans* (26). Based on transcriptional analysis, several *P*. *sojae* infection-associated bZIPs were regulated by H_2_O_2_ treatment (28). Several bZIP proteins from *P*. *infestans* were functionally characterized and only Pibzp1 was found essential for virulence (26, 29). In our study, we identified and analyzed a bZIP transcription factor, PlBZP32, which is an ortholog of *P. sojae* BZPc32 and possesses a bZIP-PAS structure.

CRISPR/Cas9-mediated gene disruption in *P. sojae* was established in 2016 (38), and our group also succeeded in disrupting *PlPAE4* and *PlPAE5* genes using this technique (39). However, we failed to knock out *PlBZP32* in *P*. *litchii*, suggesting that it might be an essential gene. Therefore, we investigated the function of *PlBZP32* by a gene silencing strategy. We obtained only 3 *PlBZP32*-silenced transformants out of 182 transformants screened by antibiotic resistance. The silencing efficiency of this gene is much lower than that of *PlMAPK10*, as reported by our group previously (40). Out of all the G418-resistant transformants, only around 37% contain the empty vector, based on PCR verification. This gene was downregulated by 31% to 39% in the mycelia of the three *PlBZP32*-silenced transformants and 65% to 87% in the zoospores. The efficiency of downregulating this gene expression seems higher in zoospores than in mycelia, likely because the transcriptional level of *PlBZP32* is highly expressed in zoospores compared to mycelia. The gene silencing strategy might affect other genes with high identity in the sequence. However, other *P*. *litchii* BZP-encoding genes showed less than 51% identity with *PlBZP32*; thus, we assumed that our targeted silencing of *PlBZP32* may not affect the expression of other BZP-encoding genes. On the other hand, homology-based transcriptional silencing may also cause off-target effects on the neighboring gene(s) (32, 33), but we managed to confirm that it did not happen to the annotated gene *Pl105397* that was proximal to the *PlBZP32* locus.

The production of sporangia and germination of cysts are very important for the asexual reproduction, dissemination, and infection of *P*. *litchii*. In this study, the *PlBZP32*-silencing mutants produced more sporangia but with reduced germination rate of cysts, and the sporangial germination rate was not affected by silencing of *PlBZP32*. These results suggested that *PlBZP32* might be a negative regulator in the production of sporangia and a positive regulator in the germination of cysts.

We further found that PlBZP32 is required for the full virulence of *P*. *litchii* and its resistance to oxidative stress caused by H_2_O_2_. This may indicate that PlBZP32 regulates *P*. *litchii* virulence via resistance to oxidative stress. In supporting this hypothesis, we found that the enzymatic activities of ROS scavenger peroxidases and laccases were both decreased in the *PlBZP32*-silencing mutants. This finding is consistent with the report of *M*. *oryzae* MoAP1 (14). However, the direct target genes of the PlBZP32 transcription factor in regulating oxidative resistance and/or pathogenicity of *P*. *litchii* are unclear.

PAS domain-containing proteins detect a wide range of physical and chemical stimuli and associate with a series of signal transduction systems (41). PlBZP32 contains a PAS domain immediately following the bZIP domain, suggesting that it might interact with some small ligand through a different pathway than other bZIP transcription factors. Further study is needed for characterizing the function of the PAS domain in bZIP transcription factors.

Overall, our study identified a bZIP transcription factor responsible for *P. litchii* development, oxidative stress response, and pathogenicity. At present, we do not fully understand its regulatory mechanism, particularly the function of its PAS domain (and the corresponding ligands/signal molecules) and its downstream target genes. It is also worth further investigating the function diversity (if any) among oomycete species.

## MATERIALS AND METHODS

### Bioinformatics analysis.

The *P. litchii BZP32* (GenBank accession number MT396989) sequence was obtained by a BLAST search using *PsBZPc32* as a bait in NCBI (BioProject ID PRJNA290406) (30). Sequences of BZPc32 orthologs in *P. sojae* (v1.1), *P. ramorum* (v1.1), *P. capsici* (v11.0), *P. infestans*, and *T. pseudonana* (v3.0) genomes were obtained from the DOE Joint Genome Institute (JGI) database (genome.jgi.doe.gov), and the BZP32 ortholog in *Pythium ultimum* was obtained from the *Pythium* Genome Database (pythium.plantbiology.msu.edu). Orthologs of PlBZP32 in *P. parasitica* and *Albugo candida* were obtained from the NCBI database (https://www.ncbi.nlm.nih.gov/). All of these sequences were listed in Table S2. The sequence alignment (Muscle algorithm) and phylogenetic tree (neighbor-joining algorithm with 1,000 bootstrap replications) were constructed using the MEGA7 program (http://megasoftware.net).

10.1128/mSphere.00261-20.2 TABLE S2 Sequences of orthologs of PlBZP32 used in this study. Download Table S2, XLSX file, 0.01 MB. Copyright © 2020 Kong et al. 2020 Kong et al. This content is distributed under the terms of the Creative Commons Attribution 4.0 International license.

### *P. litchii* strain and culture conditions.

*P*. *litchii* strain SHS3 (wild type) was isolated from Guangdong Province, China, and cultured on carrot juice agar (CJA) medium (juice from 200 g carrot for 1 liter medium, 15 g agar/liter for solid media) at 25°C in the dark. The *PlBZP32*-silenced transformants were maintained on CJA medium containing 50 μg/ml G418.

### Analysis of transcriptional profile of *PlBZP32*.

Total RNA was extracted using the total RNA kit (catalog [cat.] number R6834-01; Omega,). Samples included mycelia, sporangia, zoospores, cysts, germinating cysts, oospores, and mycelial mats inoculated on expanded tender lychee leaves of 8 to 10 days old for 1.5, 3, 6, 12, and 24 h (40, 42). The first-strand cDNA was synthesized from total RNA by oligo(dT) priming using a Moloney murine leukemia virus (MMLV) reverse transcriptase kit (number S28025-014; Invitrogen, Carlsbad, CA). The transcription profile of *PlBZP32* was analyzed with qRT-PCR, with *ACTIN* (GenBank accession number MT396988) as an internal control. The primers pairs PlBZPRTF (CCTCTGCGGTGTTCCTTTTC) and PlBZPRTR (CGTGTTGAGCTTCGTGAACA); and PlActF (TCACGCTATTGTTCGTCTGG) and PlActR (TCATCTCCTGGTCGAAGTCC) were used to amplify *PlBZP32* and *ACTIN*, respectively. The relative fold change was calculated using the threshold cycle (2^−ΔΔ^*^CT^*) method (43).

Sporangia were harvested by ﬂooding the mycelia, which had been cultured on CJA media for 5 days, with sterile water, then ﬁltering the subsequent suspension through a 100-μm strainer (44). The suspension was incubated with sterile distilled water at 16°C for 2 h for releasing zoospores. For oospore collection, *P*. *litchii* strains were inoculated on CJA medium which was covered by an Amersham Hybond membrane. After 10 days, the aerial mycelia were separated from the medium by removing the Amersham Hybond membrane. Then, oospores were scraped from the medium surface and collected.

### Transformation of *P. litchii*.

The full-length open reading frame of *PlBZP32* was amplified with primers PlBZP32-*Bsi*wI-F (AAACGTACGATGGACTTCACGTCGCCTAATG) and PlBZP32-ClaI-R (AAAATCGATAATGTCGCGGTCCACAC), using the cDNA from *P. litchii* strain SHS3 as the template. The amplified *PlBZP32* fragment was ligated into the pTORmRFP4 vector digested with ClaI and BsiWI in antisense orientation (45, 46).

*PlBZP32*-silenced mutants were generated using an established protocol (44). Preliminary transformants were screened on CJA media containing 50 μg/ml G418. PCR screening for *PlBZP32-*silencing transformants was performed with primers pHAM34-F (GCTTTTGCGTCCTACCATCCG) and PlBZP32-BsiWI-F. The gene silencing efficiency was evaluated with real-time RT-PCR, using the primers, PlBZPRTF/PlBZPRTR and PlActF/PlActR, as listed above.

### Pathogenicity assays on lychee leaves.

The 8- to 10-day-old soft expanded lychee leaves were collected from the same plant in an orchard in South China Agricultural University, Guangzhou, Guangdong Province, China. The 5-mm hyphal plugs of the *PlBZP32*-silenced mutants were inoculated on the abaxial side of lychee leaves in a dark box and then were placed in climate room under 80% humidity in the dark at 25°C for 36 h (44). The virulence of each transformant was tested with the wild-type strain (SHS3) and CK strain as positive controls. Lesion length (the longest diameter of the lesion) was measured at 36 h postinoculation. At least three independent repeats were performed for each instance with 12 leaves/repeats.

### Extracellular enzyme activity assays.

The detection of peroxidase secretion and laccase activity was performed following the reported procedure (37). The experiments were repeated three times independently, with three technical replicates each time.

### Data availability.

Sequences of *P. litchii ACTIN*, *PlBZP32*, and *Pl105397* were submitted to GenBank under accession numbers MT396988, MT396989, and MT396990, respectively.
