# Supplementary figures and images for: The Basic Leucine Zipper Transcription Factor PlBZP32 Associated with the Oxidative Stress Response Is Critical for Pathogenicity of the Lychee Downy Blight Oomycete Peronophythora litchii
Source: mSphere. 2020 Jun 3;5(3):e00261-20. doi: 10.1128/mSphere.00261-20 (PMC7273347; doi:10.1128/mSphere.00261-20)

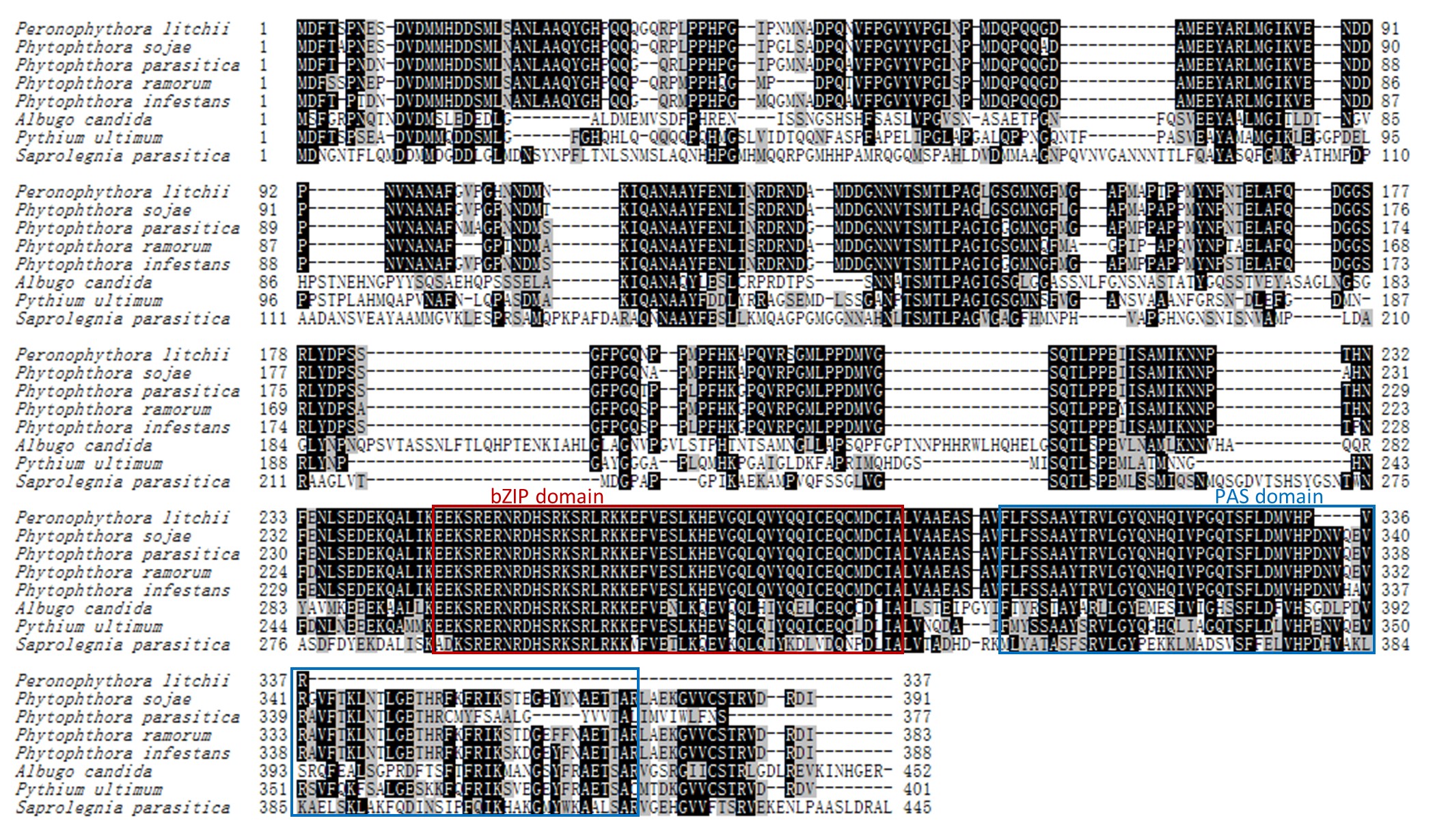

Supplement: FIG S1 [file mSphere.00261-20-sf001.jpg]

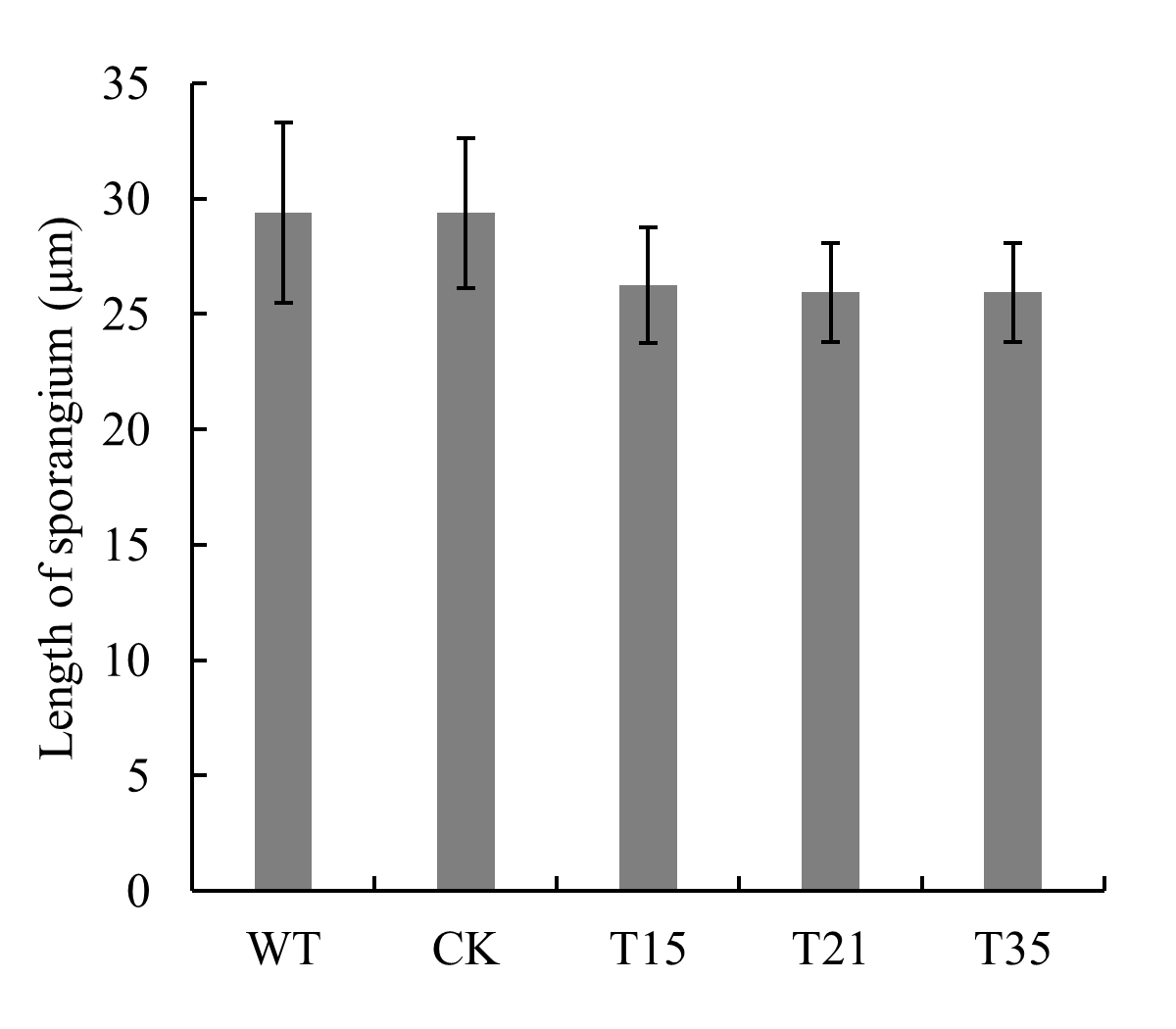

Supplement: FIG S2 [file mSphere.00261-20-sf002.tif]

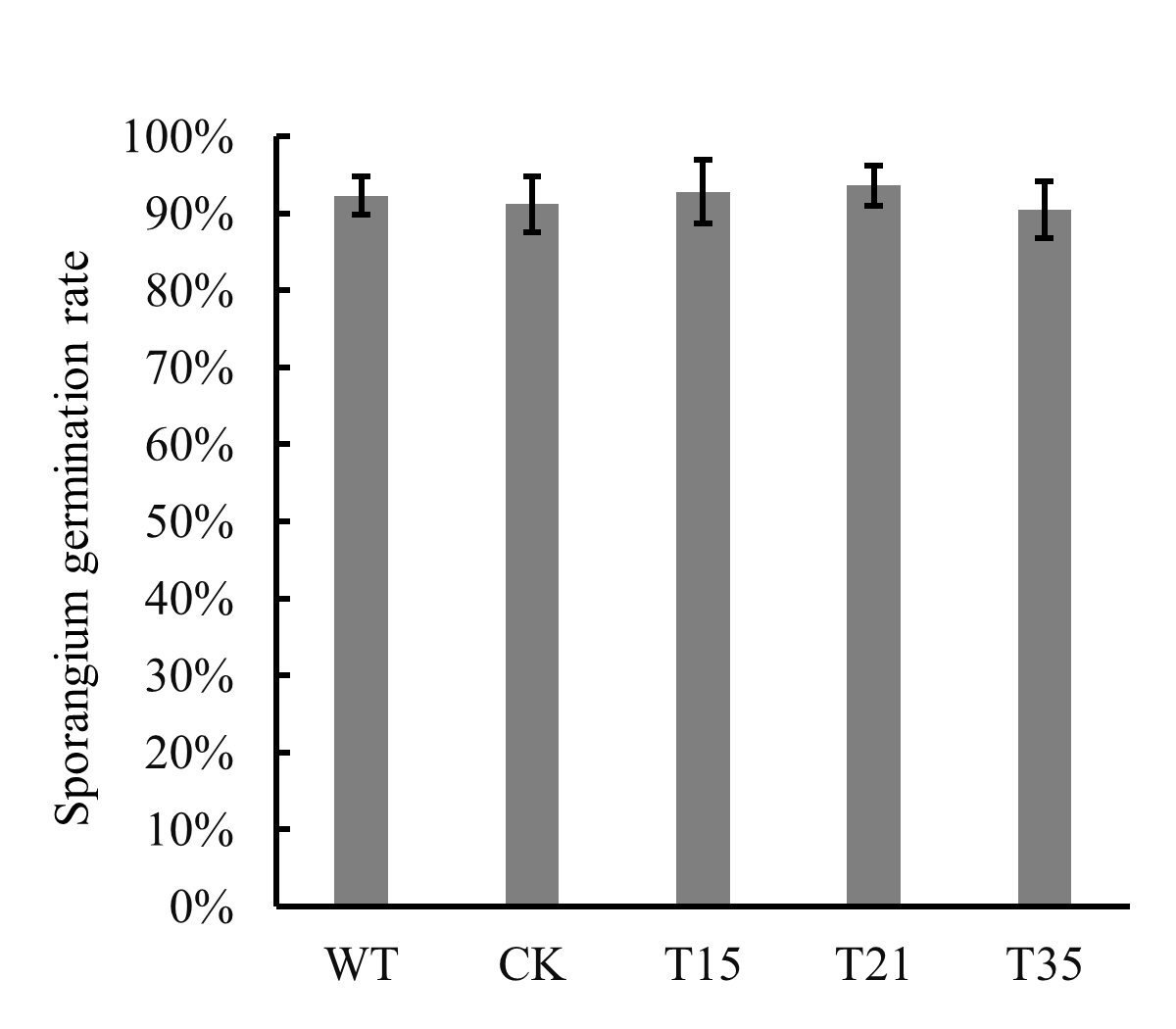

Supplement: FIG S3 [file mSphere.00261-20-sf003.tif]
